# Transport Inhibition of Digoxin Using Several Common P-gp Expressing Cell Lines Is Not Necessarily Reporting Only on Inhibitor Binding to P-gp

**DOI:** 10.1371/journal.pone.0069394

**Published:** 2013-08-16

**Authors:** Annie Albin Lumen, Libin Li, Jiben Li, Zeba Ahmed, Zhou Meng, Albert Owen, Harma Ellens, Ismael J. Hidalgo, Joe Bentz

**Affiliations:** 1 Department of Biology, Drexel University, Philadelphia, Pennsylvania, United States of America; 2 Preclinical Drug Metabolism and Pharmacokinetics, GlaxoSmithKline, King of Prussia, Pennsylvania, United States of America; 3 Absorption Systems LLC, Exton, Pennsylvania, United States of America; Griffith University, Australia

## Abstract

We have reported that the P-gp substrate digoxin required basolateral and apical uptake transport in excess of that allowed by digoxin passive permeability (as measured in the presence of GF120918) to achieve the observed efflux kinetics across MDCK-MDR1-NKI (The Netherlands Cancer Institute) confluent cell monolayers. That is, GF120918 inhibitable uptake transport was kinetically required. Therefore, IC_50_ measurements using digoxin as a probe substrate in this cell line could be due to inhibition of P-gp, of digoxin uptake transport, or both. This kinetic analysis is now extended to include three additional cell lines: MDCK-MDR1-NIH (National Institute of Health), Caco-2 and CPT-B2 (Caco-2 cells with BCRP knockdown). These cells similarly exhibit GF120918 inhibitable uptake transport of digoxin. We demonstrate that inhibition of digoxin transport across these cell lines by GF120918, cyclosporine, ketoconazole and verapamil is greater than can be explained by inhibition of P-gp alone. We examined three hypotheses for this non-P-gp inhibition. The inhibitors can: (1) bind to a basolateral digoxin uptake transporter, thereby inhibiting digoxin's cellular uptake; (2) partition into the basolateral membrane and directly reduce membrane permeability; (3) aggregate with digoxin in the donor chamber, thereby reducing the free concentration of digoxin, with concomitant reduction in digoxin uptake. Data and simulations show that hypothesis 1 was found to be uniformly acceptable. Hypothesis 2 was found to be uniformly unlikely. Hypothesis 3 was unlikely for GF120918 and cyclosporine, but further studies are needed to completely adjudicate whether hetero-dimerization contributes to the non-P-gp inhibition for ketoconazole and verapamil. We also find that P-gp substrates with relatively low passive permeability such as digoxin, loperamide and vinblastine kinetically require basolateral uptake transport over that allowed by +GF120918 passive permeability, while highly permeable P-gp substrates such as amprenavir, quinidine, ketoconazole and verapamil do not, regardless of whether they actually use the basolateral transporter.

## Introduction

It is well established that transporters play an important role in absorption, distribution, metabolism and elimination of drugs. Inhibition of drug transporters can affect drug safety and efficacy. The International Transporter Consortium published a white paper reviewing the clinically important drug transporters and summarizing which *in vitro* methods are suitable for assessing drug-drug interaction (DDI) risks [1]. P-glycoprotein (P-gp) is listed as one of the ABC transporters of emerging clinical importance. The risk for a DDI resulting from P-gp inhibition is assessed by determining the *in vitro* inhibitor concentration required to reduce probe-substrate transport by 50%, i.e. the IC_50_ [2] [3] [4] [5]. Digoxin is typically used in *in vitro* inhibition studies as a clinically relevant P-gp probe substrate since it has a narrow therapeutic window and digoxin clinical drug-drug interactions have been ascribed to P-gp inhibition. Inhibition of digoxin transport is often determined using confluent polarized cell lines expressing high levels of P-gp such as Caco-2 [2] [6] [7] [8], MDCK-MDR1-NKI (from the Netherlands Cancer Institute) [9], MDCK-MDR1-NIH (from NIH) [10] and LLC-PK1 (from the Netherlands Cancer Institute) [11].

In the past, it has been assumed that when an investigational drug inhibits transport of digoxin across these cell lines, it is due to inhibition of P-gp. However, Acharya et al. [12] found in the MDCK-MDR1-NKI cell line that digoxin is not only a substrate of P-gp, but also required both basolateral and apical uptake transport, in excess of that allowed by passive permeability in the presence of GF120918, to explain its bidirectional trans-cellular transport kinetics. Acharya et al. [12] ascribed this observation to the presence of apical and basolateral digoxin uptake transporters. These kinetically identified uptake transporters facilitate digoxin entry into the cell to gain access to the substrate binding site on P-gp. Acharya et al. [12] also found that the P-gp substrate loperamide required a basolateral uptake transporter to explain its transporter kinetics at low substrate concentrations only (0.03–1 µM), while amprenavir and quinidine did not. These results were confirmed using a much more rigorous kinetic fitting analysis in Agnani et al. [13]. Due to a production error, all of the intended µM and µL in [13] were published as mM and mL.

The putative digoxin and loperamide uptake transporters were identified kinetically by virtue of the fact that they are inhibitable by low concentrations of GF120918. Prototypical inhibitors of organic anion transporters (OATPs, OATs) and organic cation transporters (OCTs) did not affect digoxin or loperamide uptake transport, therefore the identity of the putative digoxin uptake transporter remains unknown. The existence of an as yet unidentified digoxin uptake transporter has also been proposed in Caco-2 cells [14] [15], sandwich cultured human hepatocytes [16] and HEK cells [17]. Digoxin uptake in the sandwich-cultured human hepatocytes was similarly not inhibitable by prototypical inhibitors of hepatic OATP, OAT and OCT transporters [16].

Digoxin uptake transport may have important implications for P-gp IC_50_ determinations using digoxin as probe substrate, since the observed overall IC_50_ could well be a convolution of inhibition of both uptake transport as well as P-gp. We demonstrate here by kinetic analysis that the GF120918 IC_50_ value for inhibition of digoxin transport across MDCK-MDR1-NKI cells is indeed a convolution of inhibition of P-gp and basolateral digoxin uptake transport.

The investigation into the presence of GF120918 inhibitable digoxin uptake transporters has now been extended to three additional P-gp expressing cell lines: MDCK-MDR1-NIH (National Institute of Health), Caco-2 and CPT-B2 (Caco-2 cells with BCRP knockdown). For each of cell lines, we obtained IC_50_ curves for GF120918, cyclosporine, ketoconazole, and verapamil. Kinetic analysis showed that each of these cell lines contain a basolateral digoxin uptake mechanism that was inhibited by GF120918 and cyclosporine-A.

Since we have not yet been able to identify a basolateral digoxin uptake transporter, we have examined three different hypotheses to identify the source(s) of the basolateral inhibitable digoxin uptake mechanism:

1. The cell line has a basolateral digoxin uptake transporter that is inhibited.
2. Partitioning of inhibitor into the basolateral membrane causes the membrane to become less permeable.
3. There is hetero-dimerization of digoxin with the inhibitor in the basolateral donor chamber that reduces the free digoxin concentration, thereby inhibiting digoxin uptake into the cell.

Hypothesis 3, the consideration of a physical interaction between inhibitor and digoxin, is based on studies of inhibitors of enzymes, primarily kinases, in high-throughput screens that found several “promiscuous” inhibitors, i.e. inhibitors that were able to inhibit multiple and quite different enzymes [18] [19] [20]. It was found that some of these “promiscuous” inhibitors could form large aggregates, >1 µm in diameter, that appear to adsorb the enzymes onto the aggregate surface and thereby inhibit enzyme activity. In these enzyme inhibition studies three P-gp substrates were examined: digoxin, ketoconazole and nicardipine. Digoxin and ketoconazole were found not to be aggregating compounds, but nicardipine was found to make large aggregates above 30–40 µm. This system has been simplified to a model of simple hetero-dimerization, since simulations showed that both models predicted the same qualitative behavior.

In addition, we have added vinblastine to the list of drugs that kinetically need a basolateral uptake transport mechanism in the MDCK-MDR1-NKI cells. This led to the question of how small must a drug's passive permeability be in order that a kinetic need for an uptake transporter can be justified. By simulations, we have found that a kinetic need for a basolateral uptake transporter can only be observed using low concentrations of drugs with passive permeability values below about 320 nm/s, e.g. digoxin, loperamide and vinblastine. Highly permeable compounds, e.g. amprenavir, quinidine, ketoconazole and verapamil, will show no clear kinetic need for uptake transport to explain their trans-cellular transport, due to the overwhelming transport signal from passive permeability. They may access the transport mechanism, but that would not be kinetically indentifiable. This matches our observations.

## Materials and Methods

### Materials

For experiments performed at GSK (as designated in Figure Legends) materials were obtained from the following sources. Amprenavir and GF120918 were from GlaxoSmithKline (Uxbridge, Middlesex, UK); verapamil hydrochloride, vinblastine sulfate, ketoconazole and cylcosporin A were from Sigma (St. Louis, MO). ^3^H-verapamil (80 Ci/mmol) was from Perkin Elmer. ^3^H-ketoconazole (10 Ci/mmol) and ^3^H-vinblastine (20 Ci/mmol) were from American Radiolabelled Chemicals Inc. (St. Louis, MO). Dimethyl sulfoxide (DMSO) was from Sigma-Aldrich (St. Louis, MO). Dulbecco's modified Eagle's medium (DMEM) with 25 mM *N*-2-hydroxyethylpiperazine-*N*′-2-ethanesulfonic acid (HEPES) buffer, high glucose (4.5 g/L), L-glutamine, without sodium pyruvate, and with phenol red was from Invitrogen (Carlsbad, CA). The same medium without phenol red was used for transport experiments. Transwell 12-well plates with polycarbonate inserts (0.4 µm pore size and 12 mm in diameter) were obtained from Costar (Acton, MA).

For the experiments performed at Absorption Systems (as designated in Figure Legends) with the three cell lines (MDCK-MDR1-NIH, Caco-2 and CPT-B2) and the four inhibitors (GF120918, CsA, Ketoconazole and Verapamil), materials were obtained from the following sources. Cyclosporin A (CsA) was obtained from CalBiochem (La Jolla, CA). Digoxin, ketoconazole, verapamil, and D-glucose were obtained from Sigma (St. Louis, MO). GF120918 was provided by GSK. Lucifer yellow (LY), HEPES, Hanks Balanced Salt Solution (HBSS), Dulbecco's Modified Eagle Medium (DMEM), Dulbecco's phosphate buffered saline (DPBS), and fetal bovine serum (FBS) were obtained from Invitrogen (Carlsbad, CA). Dimethylsulfoxide (DMSO) was obtained from EMT (Darmstadt, Germany). Penicillin-streptomycin (PEST) and trypsin were obtained from CelGro (Herndon, VA). Transwell@ plates (12 well) were purchased from Corning Incorporated (Corning, NY). EndOhm for TEER measurements was purchased from World Precision Instruments (Sarasota, FL).

### Cells and Culturing Methodology for Transport and Inhibition Studies

For transport and inhibition studies performed at GSK (as designated in Figure Legends). P-gp cell line and culture conditions have been described previously [5] [12] [21] [22]. Briefly, the Madin-Darby Canine Kidney II cell line overexpressing human MDR1 was purchased from The Netherlands Cancer Institute (Amsterdam, The Netherlands) and denoted as MDCK-MDR1-NKI. The P-gp gene is stably transfected in this cell line, i.e. no drugs were needed to select for the MDR1 expressing cells [5] [9].

Cells were split twice a week and maintained in culture media (DMEM supplemented with 10% Fetal Bovine Serum, 50 units/ml penicillin and 50 µg/ml streptomycin). Cells were kept at 37°C in 5% CO_2_. All transport and inhibition assays were performed with cells between passage 30–55. P-gp mediated transport was measured in 12-well transwell plates fitted with polycarbonate membrane inserts. Cells were seeded at a density of 175,000–200,000 cells per insert and grown for four days in culture media. Cells were given fresh media one day after seeding.

Prior to the transport experiments, culture media was removed and cells were preincubated for 30 minutes with either transport medium alone or transport medium supplemented with 2 µM GF120918 to inhibit P-gp and measure the substrate's passive permeability. For incubations in the presence of GF120918, the inhibitor was added to both chambers. In addition, 100 µM Lucifer yellow was added to the donor chamber to monitor cell monolayer integrity. ^3^H-verapamil, ^3^H-ketoconazole, ^3^H-vinblastine or ^3^H-cyclosporin was added to each respective drug concentration to allow quantitation of transport from donor to receiver chambers in both directions over time, i.e. apical to basolateral (A>B) and basolateral to apical (B>A) in the presence and absence of GF120918. At the indicated time points, 25 µL samples were taken from both these chambers and counted by TopCount Model 9912.

For experiments performed at Absorption Systems LP (as designated in Figure Legends). Madin-Darby Canine Kidney cells overexpressing human MDR1 were obtained from the NIH (MDCK-MDR1-NIH), Caco 2 cells were from the ATTC and CPT-B2 cells were generated at Absorption Systems by knocking down the BCRP gene in Caco-2 cells (unpublished). Cell monolayers used in this study were prepared in accordance with the standard operating procedures (SOPs) of Absorption Systems. Briefly, stock MDCK-MDR1-NIH, Caco-2 and CPT-B2 cells were cultured in Dulbecco's modified essential medium supplemented with 10% fetal bovine serum, 1 mM sodium pyruvate, 100 µM non-essential amino acids, 4 mM L-glutamine, 100 U/mL penicillin, and 100 µg/mL streptomycin. For MDCK-MDR1-NIH cells, colchicine (40 µg/mL) was added into the complete medium for selection of cells expressing MDR1. To maintain the properties of CPT-B2 cells, puromycin (10 µg/mL) was added to the normal growth medium. For preparation of Transwell assay plates, cells were harvested from T-75 or T-150 flasks using a solution of 0.25% trypsin and 2.21 mM EDTA (Gibco Life Technologies) and cells were seeded at a density of 60,000 cells/cm^2^ on 12-well Transwell plates containing collagen-coated, micropore (0.4 µm pore size), polycarbonate filter membranes. The monolayers were grown to confluence on the filter membranes in a humidified atmosphere containing 5% CO_2_ at 37°C. MDCK-MDR1-NIH cells were incubated for six days, while Caco-2 and CPT-B2 cells were incubated for 20 days. The culture medium was changed three times per week. Monolayer integrity was assessed by measuring transepithelial electrical resistance (TEER) using an EndOhm, and determining apparent permeability (P_app_) values of selected reference compounds (digoxin, atenolol, propranolol, and lucifer yellow).

### Liposome preparation and partitioning studies

These experiments were performed at GSK. Cholesterol and porcine brain lipids were from Avanti Polar Lipids, Inc. (Alabaster, AL). Three different liposome types were prepared to roughly mimic the lipid compositions of the relevant monolayers of the plasma membrane [21] [23], while not exceeding ternary mixtures:

1. The apical membrane outer monolayer mimic was a (1∶1∶1) mixture of phosphotidylcholine/sphingomyelin/cholesterol, denoted PC/SM/chol. The partition coefficient to these liposomes was denoted K_AO_.
2. The basolateral membrane outer monolayer mimic was a (2∶1) mixture of phosphotidylcholine/cholesterol, denoted PC/chol. The partition coefficient to these liposomes was denoted K_BO_.
3. The plasma membrane inner monolayer mimic (facing the cytosol) was a (1∶1∶1) mixture of phosphotidylserine/phosphatidylethanolamine/cholesterol, denoted PS/PE/chol. The partition coefficient to these liposomes was denoted K_PC_.

Lipid mixtures were dried into thin lipid films in round bottom flasks (Buchi Rotavapor R-200, Switzerland), and freeze-dried overnight on a Flexi-Dry MP freeze-dryer (Kinetics, USA). The lipid films were then suspended in phosphate buffered saline, pH 7.4 (Gibco) and extruded through two 0.1 µm Nucleopore membranes for at least 10 cycles using a Lipex Thermal Extruder. The sphingomyelin-containing liposomes were extruded at 65°C to maintain their liquid-disordered state during extrusion.

Partitioning of the drugs to the liposomes was determined in a 20-cell equilibrium dialyzer (Spectrum, Fl) using Spectra/Por 4 membrane with a 12–14 kD molecular weight cut off. Each half of the Teflon cells received 10 µM concentrations of the cold drug but the half cell with the liposomes (5 or 10 mM lipid) received an additional 0.25 µCi/ml of appropriate radiolabeled drug. The cells were allowed to equilibrate in a 37°C water bath. At various time points (3 hr, 6 hr and 24 hr), 25 µL samples were removed from the donor and receiver chambers, placed in a 96 well luma plate and radioactivity was measured using the TopCount. These data showed that partitioning was complete by 6 hr and independent of lipid concentration at these concentrations.

The partition coefficient for the plasma membrane inner monolayer mimic, denoted K_PC_, was calculated using the relation K_PC_ = C_L_/C_W_, where C_L_ is the mol of drug per liter of aqueous buffer and C_L_ is the mol of drug per liter of lipid in the liposomes, using the average molar volume of 1.6 µL/µmol of total lipid [24]. The partition coefficients for the other liposomes, K_BO_ and K_AO_, were calculated the same way.

### Kinetic Model of Transport across a Confluent Cell Monolayer

**Figure 1:**
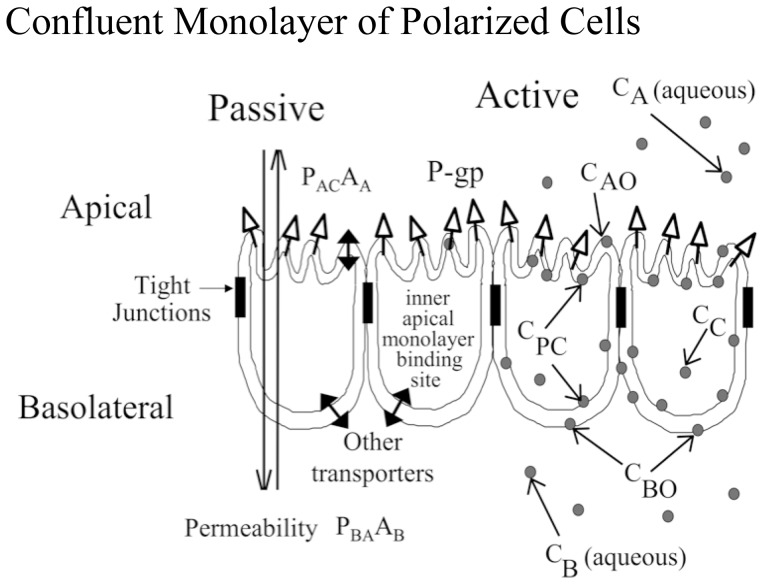
Model of a confluent cell monolayer, with the apical membrane on top and the basolateral membrane below, where it binds to the polycarbonate insert. Passive permeability occurs in both directions. P-gp expressed on the apical membrane transports substrate from the inner apical membrane monolayer into the apical chamber. The concentration of substrate in the apical and basolateral chambers, C_A_ and C_B_, are measured, while the concentration of substrate in the inner plasma membrane, C_PC_, and the cytosol, C_C_, are predicted as part of the mass action modeling and data fitting process.

Fig. 1 is a cartoon of a confluent cell monolayer, where the basolateral membrane is attached to the polycarbonate filters and P-gp (upward arrows) is expressed on the apical membrane. The apical and basolateral chambers are kept separate by the tight junctions between the cells. Active transport by P-gp is unidirectional, with substrate binding to a site on P-gp within the apical membrane inner monolayer and with efflux into the apical chamber [25] [26] [27] [28]. For many substrates, including those used in this study, passive permeability is a significant fraction of total transport and is quantitatively analyzed using the potent P-gp inhibitor, GF120918 [12] [21] [29]. While GF120918 completely inhibits P-gp, it also inhibits other transporters [12] [30].

We measure the concentration of substrate in the apical chamber, denoted C_A_, and the basolateral chamber, denoted C_B_. However, the concentration of substrate in the cytosol, denoted C_C_, and in the inner plasma membrane in contact with the P-gp binding site, denoted C_PC_, cannot be measured rigorously in real time. These intracellular and membrane concentrations are variables of the mass action model and are fitted by elementary rate constants for well-defined kinetic barriers and approximate partition coefficients according to the measured values of C_B_ and C_A_ over time [12] [13] [21].

We use the simplest competitive mass action reaction to model P-gp transport:
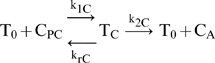
(1A)

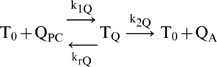
(1B)For P-gp mediated transport, Eq. (1), T_0_ is the empty transporter, C_PC_ is the substrate in the apical membrane inner monolayer, T_C_ is the transporter bound by substrate and C_A_ is the substrate after efflux into the apical chamber. For the inhibitor, Q_PC_ is the inhibitor in the apical membrane inner monolayer, T_Q_ is the transporter bound by inhibitor and Q_A_ is the inhibitor after efflux into the apical chamber. The rate constant for both probe-substrate and inhibitor are k_1_ for association with P-gp, k_r_ for dissociation from P-gp back into the membrane and k_2_ for efflux into the apical chamber. The concentrations of C_PC_ and Q_PC_ are predicted from the fitted cytosolic concentrations C_C_ and Q_C_ times the partition coefficients measured using the liposomes, respectively, Table 1 in results.

**Table 1:** Molar Partition Coefficients to 0.1 µm Extruded Unilamellar Liposomes.

Compounds tested	Apical	Basolateral	Plasma
	Outer Leaflet PC/SM/Chol (1∶1∶1) (mols)	Outer Leaflet PC/Chol (2∶1) (mols)	Cytosolic Leaflet PE/PS/Chol (1∶1∶1) (mols)
Quinidine	100±5	100±5	350±40
Amprenavir	150±15	200±10	100±2
Loperamide	300±50	450±50	1500±250
Digoxin	100±15	100±2	100±10
Verapamil	200±20	300±20	650±100
Ketoconazole	400±1	400±5	1000±60
Vinblastine	200±3	200±20	200±50
Cyclosporine	200±70	300±70	600±150

Molar concentration of drug in the bilayer (mol per L of lipid) divided by the molar concentration of drug in the aqueous buffer.

For transport mediated by other digoxin transporters, shown to exist in MDCKII-MDR1-NKI cells [12], we used facilitated transporter models, denoted as BT, for basolateral digoxin uptake transporter, and AT, for apical digoxin uptake transporter, Eq. (2). There could be more than one transporter involved at these membranes and then we would be fitting some weighted average.
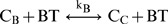
(2A)

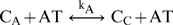
(2B)A single bidirectional first-order kinetic clearance characterizes each transporter. It was modeled as a simple facilitated transporter, to allow the mass action kinetics a degree of freedom to determine its directionality based on the fits. The fits are consistent with bidirectional or an active importer [13].

The mass action kinetic equations generated by these reactions are shown in Appendix S1 in File S1. In addition, the passive permeability contributions to changes in compartment concentrations are also included in these equations. As opposed to subtracting the +918 transport from the total transport to estimate the P-gp mediated transport, which does not account for back flow of drugs, we include the passive permeability coefficients over time within the mass action differential equations [21] [22]. The passive permeability coefficients were fixed by the time-dependent +GF120918 experiments and the partition coefficients were fixed at the values reported in Table 1. The differential equations were solved in MATLAB 2011b using the ODE23s solver, for the most stiff differential equations [21].

### Parameter Optimization

Kinetic parameters were optimized using the Particle Swarm algorithm [13], which allows fitting for any subset of the ensemble of kinetic parameters and any combination of data sets. For the MDCK-MDR1-NKI cells, the values for the efflux active P-gp surface density, T(0), and for the association rate constant, k_1_, fitted in Agnani et al. [13] were used. For the other cell lines, k_1_ remained fixed at the same value, since the cells have human P-gp and we have found for these drugs that k_1_ is essentially the same [13] [21]. For amprenavir, quinidine and loperamide we have since confirmed that k_1_, k_r_ & k_2_ are essentially the same for Caco-2 and MDCK-MDR1-NKI cells (Meng, Ellens and Bentz, unpublished). For each cell line, T(0) was fitted as a consensus for all the data. The observed cell line dependent inhibition was due to differences in P-gp inhibition and the inhibition of the other digoxin uptake transport mechanism. The goodness of fits in the model was determined using coefficient of variation calculated between model simulations and the experimentally measured kinetic data.

## Results

### Kinetic rate constants for GF120198, cyclosporine-A, ketoconazole and verapamil transport by P-gp

**Figure 2:**
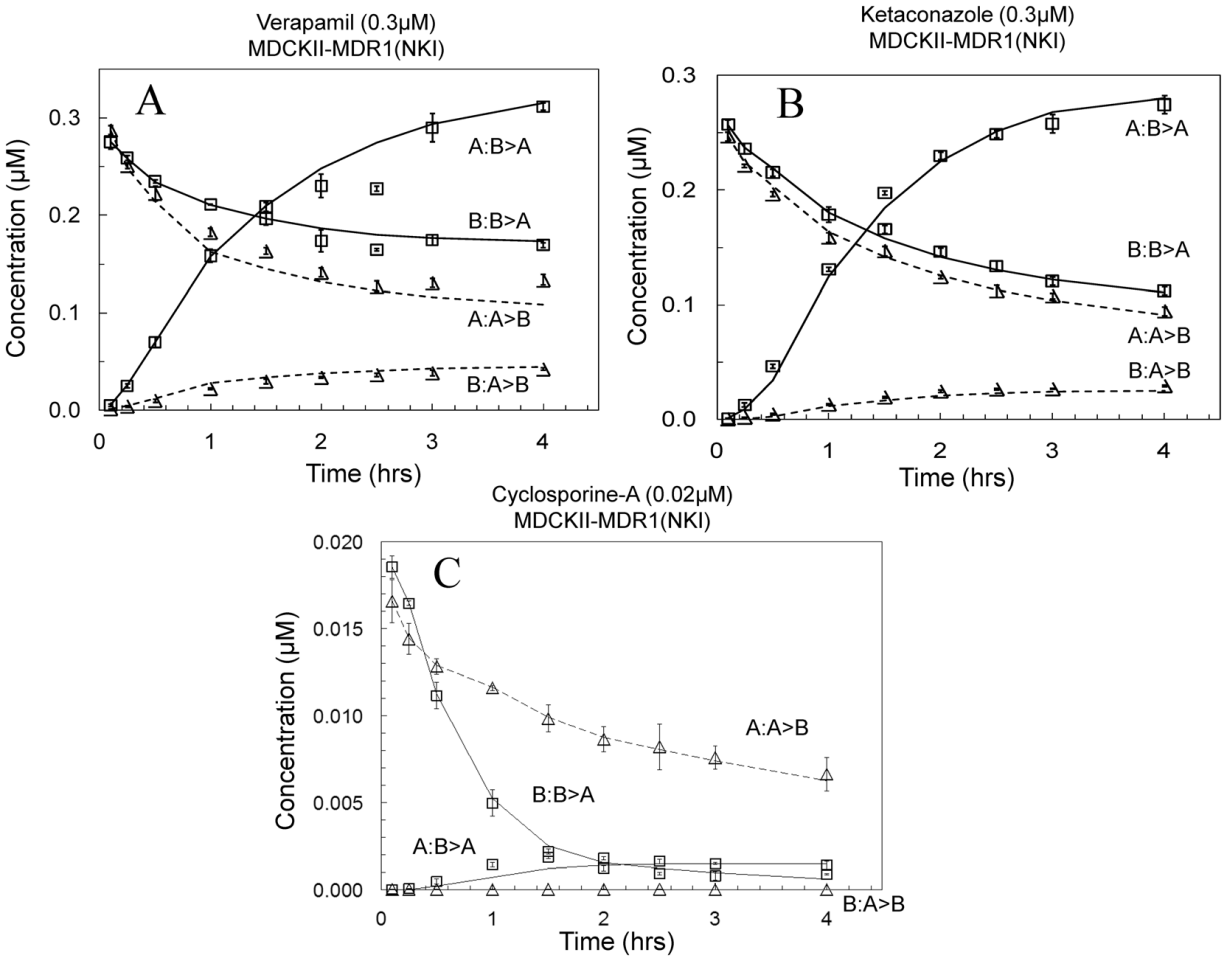
Transport data of various P-gp substrates and their fitted curves across the MDCK-MDR1-NKI cell monolayers, data acquired at GSK. Fig. 2A, 0.3 µM verapamil (VRP); Fig. 2B, 0.3 µM ketoconazole (KCZ); and Fig. 2C, 0.02 µM cyclosporine-A (CsA). The B∶B>A and A∶B>A denotes the concentration of drug in the basolateral chamber and the apical chamber respectively, when the donor chamber was the basolateral chamber, i.e., transport runs B>A. Similar nomenclature is followed for the A>B transport curves. All B>A data are shown by squares and solid lines. All A>B data are shown by triangles and dashed lines. Data shown are the average of triplicates, with standard deviation error bars. The fitted parameters are shown in Table 2.

In order to analyze the digoxin transport inhibition studies by GF120918, cyclosporin-A (CsA), ketoconazole and verapamil, as described below, we first needed to fit the basic kinetic rate constants for transport of these drugs, so we can calculate their inhibition constants for Pgp. To do this, we performed a series of bidirectional transport studies with radiolabelled verapamil and ketoconazole (initial donor chamber concentrations of 0.003, 0.01, 0.03, 0.1, 0.3, 1, 3, 10, 30 µM) and CsA (initial donor chamber concentrations of 0.01, 0.02, 0.05, 0.1, 0.2, 0.3 µM). The smaller CsA concentrations were used since CsA is a potent inhibitor of P-gp. The concentration-time profile for the donor and receiver chambers in both the basolateral to apical and apical to basolateral transport direction for each P-gp substrate was then fitted to the mass action model using the recently published improved fitting algorithm optimized for simultaneous fitting of all substrate concentrations [13]. Figs. 2A–C show the experimentally measured data (symbols) and fits to the data (lines) for a representative concentration of verapamil (VRP), ketoconazole (KCZ) and cyclosporine-A (CsA).

As shown in the mass action kinetic equations in Appendix S1 in File S1, the fitting of the transport kinetics only gives us the product of K_PC_K_C_, where K_PC_ is the partition coefficient of the drug to the cytosolic face of the plasma membrane and K_C_ is the binding constant of the drug to P-gp from the plasma membrane. The estimate of the partition coefficient of a drug into the inner apical monolayer allows an estimate of the binding constant to P-gp from the lipid membrane.

Partition coefficients for amprenavir, cyclosporin-A, digoxin, ketoconazole, loperamide, quinidine, verapamil and vinblastine were measured using 0.1 µm extruded unilamellar liposomes for three different lipid compositions that mimic roughly the lipids in plasma membrane monolayers: (1) apical outer monolayer PC/SM/chol (1∶1∶1) mols; (2) basolateral outer monolayer PC/chol (2∶1) mols; and (3) inner cytosolic monolayer PS/PE/chol (1∶1∶1) mols [21].

While these liposome compositions are simple compared to the cell membranes, they give us a reasonable measure of the independent contributions to drug transport by the drug partitioning into the membrane and by the drug binding to P-gp from within the membrane. The cells cannot be used directly for these measurements, since the drugs are adequately permeable to allow binding throughout the cell for any defensible equilibrium measurement [21].

Table 1 shows the measured partition coefficients for all drugs used in this study except GF120918 since it is not available in radiolabelled form and nicardipine that was only used as an inhibitor in this study. Amprenavir and quinidine partitioning was basically the same as measured previously in Tran et al. [21], while loperamide partitioning into the cytosolic monolayer composition was about half that measured previously. The reason for the difference is not known.

Verapamil, ketoconazole and CsA data were well fitted using only the drug specific P-gp rate constants k_r_ (dissociation from P-gp to the membrane) and k_2_ (efflux from P-gp into the apical chamber), along with the drug-independent rate constant k_1_ (association to P-gp from the membrane) and the efflux active P-gp surface density for these cells, Table 2. The fit for CsA transport shown in Fig. 2C required that CsA binding to P-gp was essentially irreversible, k_r_∼1 s^−1^, and efflux was very slow, k_2_∼0.01 s^−1^, Table 2. The extent of CsA transport was too small to allow testing of whether CsA had an uptake mechanism similar to digoxin.

**Table 2:** Fitted Consensus Kinetic Values for MDCK-MDR1-NKI Confluent Cell Monolayers.

Substrate	Association to P-gp	Efflux Active	Dissociation from P-gp	Efflux from P-gp into Apical Chamber	Binding Constant to	Passive Permeability Coefficient at steady-state^f^	
	k_1_ (M^−1^s^−1^)^a^	P-gp Surface Density [P-gp] (per µm^2^)^b^	into Apical Bilayer k_r_ (s^−1^)^c^	k_2_ (s^−1^)^d^	P-gp from Inner Apical Membrane K_C_ (M^−1^)^e^ = k_1_/k_r_	P_BA_ P_AB_ (nm/sec) Other Transporters k_B_ (s^−1^) k_A_(s^−1^)	
Amprenavir (n = 19)	(1±0.4)×10^+8^	800±200	(7±3)×10^+4^	30±8	(1±0.2)×10^+3^	420±50 0	350±30 0
Digoxin (n = 4)	Same as above	Same as above	(3±1)×10^+4^	3±1	(3±0.3)×10^+3^	50±10 40±3	40±10 40±20
Loperamide (n = 31)	Same as above	Same as above	(2±1)×10^+4^	0.4±0.08	(5±0.4)×10^+3^	320±90 100±7	320±70 0
Quinidine (n = 16)	Same as above	Same as above	(4±2)×10^+3^	3±0.4	(3±0.6)×10^+4^	670±50 0	670±50 0
Verapamil (n = 8)	Same as above	Same as above	(2±1)×10^+4^	0.1±0.02	(5±0.2)×10^+3^	580±160 0	540±60 0
Ketoconazole (n = 8)	Same as above	Same as above	(3±1.5)×10^+4^	0.2±0.1	(3±0.1)×10^+3^	680±160 0	730±70 0
Vinblastine (n = 8)	Same as above	Same as above	(5±2.5)×10^+4^	3±0.5	(2±0.4)×10^+3^	90±10 60±5	55±20 0
Cyclosporin-A (n = 8)	Same as above	Same as above	∼1	∼0. 01	∼1×10^+8^	150±50 0	25±20 0
GF120918 (n = 8)^g^	Same as above	Same as above	∼1	∼0.01	∼1×10^+8^	“500”^g^ 0	“500”^g^ 0

a k_1_ is the drug independent association rate constant from the membrane to P-gp. The average value from Agnani et al. [13] is shown. This value is fixed for all drugs fitted here.

b T(0) is the surface density of efflux active P-gp in the apical membrane inner monolayer for all drugs. The average value from Agnani et al. [13] is shown. This value is fixed for all drugs fitted here. The units P-gp/µm^2^ can be converted to µmols P-gp per liter of inner apical membrane simply by dividing by 0.8, e.g. 800 P-gp/µm^2^ is equal to 1×10^−3^ M P-gp in the inner apical leaflet [21].

c k_r_ is the dissociation rate constant from the P-gp binding site into the apical bilayer.

d k_2_ is the efflux rate constant from the P-gp binding site into the apical chamber.

e K_C_ = k_1_/k_r_ is the substrate binding constant from inner apical membrane monolayer to P-gp.

f P_BA_ and P_AB_ refers to the lipid bilayer steady-state passive permeability coefficient, B>A and A>B respectively, measured +GF120918. k_B_ and k_A_ refers to the 1^st^ order clearances for transport through bidirectional uptake transporters.

g It was assumed that the partition coefficient was the same as CsA and that the permeability coefficients were 500 nm/s, since they could not be measured, due to the lack of radiolabelled GF120918. The chosen values will not have a significant effect upon the results with GF120918 as an inhibitor.

We could not look at transport of GF120918, because no radiolabelled compound was available. As a kinetic surrogate, we used the inhibition of amprenavir transport by GF120918. Since amprenavir has no kinetic need for other transporters besides P-gp [12] [13] [21], GF120918 binding to P-gp will be completely characterized by the inhibition of amprenavir transport. Using 0.03 µM amprenavir as substrate and eleven concentrations of GF120918, ranging from 0.002–2 µM for inhibition of amprenavir transport, we found that GF120918 binding was essentially irreversible, i.e. k_r_∼1 s^−1^ and efflux was very slow, k_2_∼0.01 s^−1^ (data not shown).

Additionally, these fits assumed that the partition coefficients of GF120918 were the same as those of CsA, Table 2, since we could not measure them directly. Simulations depend only upon the ratio of k_r_/K_PC_, with respect to these two parameters, and fits have shown that the ratio of k_r_/K_PC_ is essentially constant. Fits for k_2_ are essentially unaffected by changes in the partition coefficient. So our conclusions here will not change once the partition coefficients for GF120918 are known. The permeability coefficients were set to 500 nm/s, but these parameters were only relevant for the inhibition studies described below, where GF120918 was added to both chambers for 30 min, so equilibrium binding could be assumed [12]. Provided that the permeability coefficients are larger than about 320 nm/s, they have minimal effect on the fitted kinetic values for GF120918.

### Inhibition of digoxin transport by GF120918 in MDCK-MDR1-NKI cells

Since digoxin is a substrate of uptake and efflux transport in MDCK-MDR1-NKI cells [12] [13] and since the uptake transport is inhibitable by GF120918, inhibition of B>A digoxin transport across these cells is expected to be due to inhibition of P-gp, the basolateral uptake transporter or both. Using: (1) the mass action kinetics derived elementary rate constants for binding of digoxin to P-gp, Table 2; (2) the association constant (K_QB_) of the inhibitor to the basolateral uptake transporter inhibiting the digoxin uptake clearance, k_B,_ Eq. 3, shown below; and (3) the elementary rate constants for binding of GF120918 to P-gp described above, the contributions of P-gp inhibition versus the inhibition of the uptake transporter to the overall digoxin transport inhibition can be deconvoluted.

**Figure 3:**
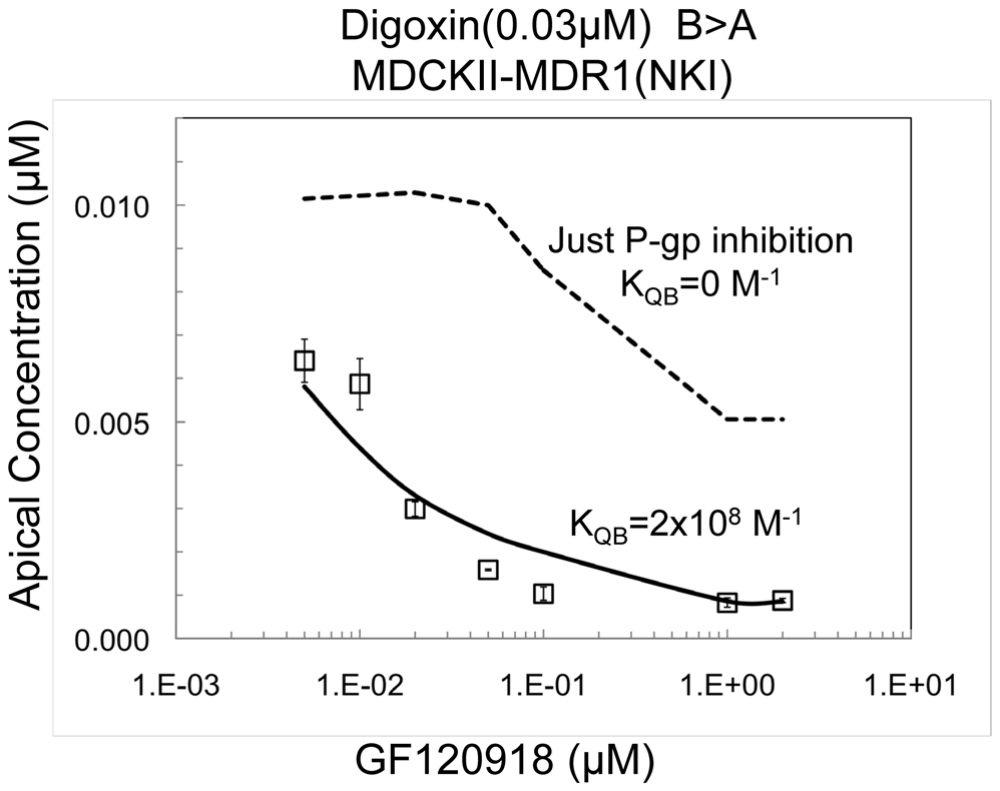
Inhibition of 0.03 µM digoxin B>A transport at 2 hr across the MDCK-MDR1-NKI cell monolayer with increasing concentrations of GF120918, data acquired at GSK. Data shown are the average of triplicates, with standard deviation error bars. The dashed line shows the predicted inhibition when GF120918 only binds to P-gp, i.e. not to the basolateral digoxin uptake transporter. The solid line shows the fit when GF120918 binds to P-gp and the basolateral digoxin uptake transporter. K_QB_ is the effective association constant of GF120918 to the basolateral digoxin uptake transporter defined by Eq. (3), Table 3. K_QB_ combines the uptake transporter surface density, the binding constant of the inhibitor to the uptake transporter and, if the inhibitor accesses the binding site from the membrane, the inhibitor partition coefficient.

The inhibition of 0.03 µM digoxin B>A transport across MDCK-MDR1-NKI confluent cell monolayers by GF120918 (0.005–2 µM) was measured, Fig. 3. The IC_50_ was in the range of 0.01–0.02 µM GF120918, which agrees with [4]. The dashed line shows the transport inhibition curve predicted from the best fit for the kinetic parameters shown in Table 2, assuming that GF120918 only inhibits P-gp and not digoxin uptake transport.

Clearly, the predicted inhibition of P-gp underestimates the measured GF120918 inhibition at all concentrations. The level of inhibition at all GF120918 concentrations was much larger than possible based on P-gp inhibition alone, showing substantial non-P-gp inhibition. The question is: what is the mechanism of this inhibition of digoxin uptake transport?

### Mechanism of basolateral digoxin uptake transport and uptake transport inhibition

Hypothesis 1 was that the non-P-gp inhibition could be due to the binding of the inhibitor to the basolateral digoxin uptake transporter. The first order basolateral uptake clearance for digoxin in the MDCK-MDR1-NKI cells is denoted k_B_, Table 2. We propose the simplest binding relationship for modeling the inhibition of this uptake clearance:
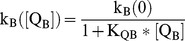
(3)where [Q_B_] is the inhibitor concentration in the basolateral donor chamber and k_B_([Q_B_]) is the basolateral uptake clearance of digoxin in the presence of the inhibitor. Eq. 3 is equivalent to the competitive Michaelis-Menten equation when the substrate Km is much larger than the substrate concentration. We do not have enough data to fit two independent parameters, so K_QB_ combines both uptake transporter surface density and its binding constant to the inhibitor. If the inhibitor accesses the basolateral transporter binding site from the membrane, then K_QB_ also embeds the partition coefficient of the inhibitor to the outer basolateral monolayer. Thus, for a given inhibitor, the K_QB_ values for an inhibitor across different cell lines cannot be compared.

In Fig. 3, there was essentially no fit when only P-gp was inhibited, i.e. when K_QB_ = 0, dashed line. The solid line is the predicted inhibition assuming inhibition of both P-gp and the uptake transporter when K_QB_ = 2×10^8^ M^−1^ for GF120918. All other kinetic parameters were fixed by the values in Table 2. The fit is reasonable.

Hypothesis 2 was that the non-P-gp inhibition could be due to the partitioning of the inhibitor into the basolateral membrane and reducing the digoxin passive permeability by some mechanism. While partitioning would be linear with inhibitor concentration, a mechanism of permeation reduction could be non-linear with inhibitor concentration, e.g. sigmoidal. The mol fraction of inhibitor in the lipid bilayer X_Q_ is defined as the mols of inhibitor in the bilayer divided by the total number of mols of lipid and inhibitor in the bilayer. It depends upon the concentration of inhibitor in the basolateral compartment, [Q_B_], the partition coefficient of the inhibitor into the basolateral outer membrane, K_BO_, and the average molar volume of the basolateral outer monolayer V_mol_≈1.6 L/mol [24].
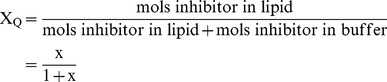
(4)where x = V_mol_K_BO_[Q_B_].

Fig. 3 shows substantial non-P-gp inhibition at 0.1 µM GF120918. With [Q_B_] = 0.1 µM GF120918, if we assume a very high partition coefficient of GF120918 into the outer basolateral membrane of K_BO_ = 1×10^4^, which is more than 20-fold higher than any K_BO_ observed value thus far (Table 1), then x = 1.6×10^−3^ and the molar ratio of GF120918 to lipid of roughly X_Q_ = 1.6×10^−3^, or fewer than 2 molecules of GF120918 for every 1000 molecules of total lipid. Such a miniscule amount of GF120918 is unlikely to affect membrane permeability by any known mechanism, thus hypothesis 2 was unlikely for GF120918. Hypothesis 3 was that hetero-aggregation of digoxin with GF120918 in the basolateral chamber results in a reduced free digoxin concentration, thereby reducing passive permeability. This is a simplified model of extensive work showing that at least one P-gp substrate, nicardipine, can form micron sized aggregates [18] [19] [20]. Theoretically such colloidal aggregates could partition digoxin, thereby reducing the free digoxin concentration in the donor chamber.

We used a simpler model of hetero-dimerization between digoxin and the inhibitor, rather than dealing with an aggregate size of inhibitor and a partition coefficient for digoxin into that aggregate. Both models yield the same qualitative behavior (simulations not shown). So, we have added the following equilibrium reaction to our standard mass action kinetic model for P-gp transport [13], i.e. this equilibration happens at every numerical integration step:
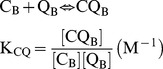
(5)where C_B_ is the probe-substrate, e.g. digoxin, Q_B_ is the inhibitor and C_QB_ is the heterodimer of digoxin-inhibitor in the basolateral chamber, i.e. donor chamber. We assume that this equilibrium is rapid compared to transport. We use the binding constant, K_CQ_, to titrate the reduction of free digoxin from the donor chamber by hetero-dimerization. Whether higher order aggregates form or not does not affect our conclusions about whether aggregation is a plausible mechanism to explain the non-P-gp inhibition of transport.

**Figure 4:**
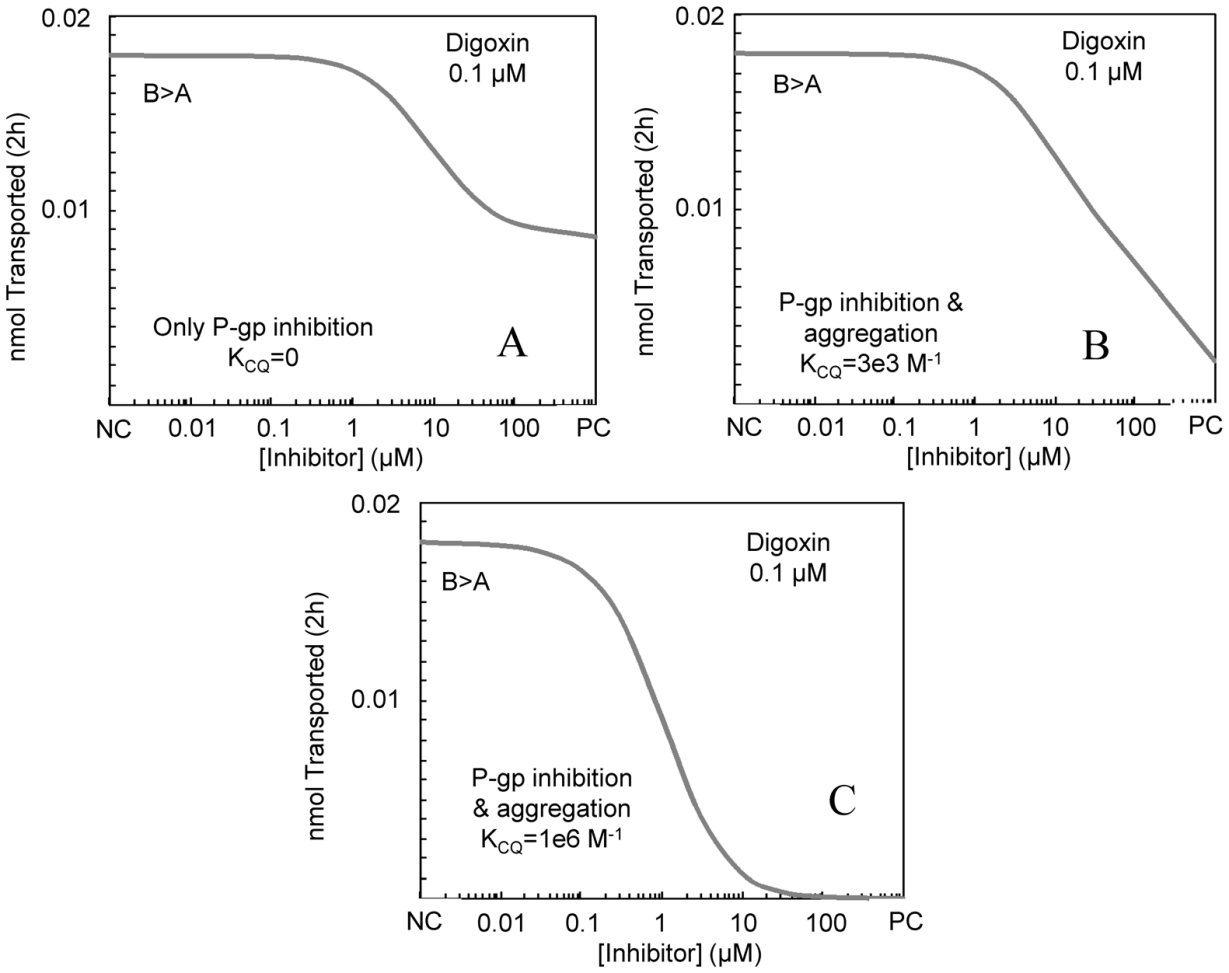
Simulated inhibition of 0.1 µM digoxin B>A transport across the MDCK-MDR1-NKI cells by an inhibitor under three scenarios of inhibitor binding to digoxin in the donor and receiver chambers. Fig. 4A shows the case where K_CQ_ = 0, i.e. digoxin transport was inhibited solely by P-gp inhibition. The curve is sigmoidal. Fig. 4B shows the case where K_CQ_ = 3e3 M^−1^, i.e. digoxin transport was inhibited by P-gp inhibition and by loss of free digoxin in the donor chamber due to heterodimerization to the inhibitor. The curve is not sigmoidal. Fig. 4C shows the case where K_CQ_ = 1e6 M^−1^, i.e. digoxin transport was inhibited by P-gp inhibition and by loss of free digoxin in the donor chamber due to strong heterodimerization to the inhibitor. The curve is sigmoidal with a large inhibitor concentration plateau, i.e. PC plateau, of no transported digoxin, since there is no free digoxin.

Fig. 4A shows the simulated digoxin transport inhibition curve with K_CQ_ = 0, i.e. no dimerization, and only inhibition of P-gp. A simple sigmoidal curve is seen with the small inhibitor concentration plateau, denoted negative control or NC plateau, showing the nmol transported after 2 h due to the combined action of digoxin's basolateral uptake transporter (k_B_ in Table 2), passive permeability and P-gp efflux kinetic parameters. The large inhibitor concentration plateau, denoted the positive control or PC plateau, is significantly larger than zero.

Fig. 4B shows the simulated digoxin transport inhibition curve by the same inhibitor with hetero-dimerization, K_CQ_ = 3×10^3^ M^−1^. The NC plateau is the same, but as the inhibitor concentration increases, a non-sigmoidal decrease of digoxin transport is observed, which may be heading toward zero nmol transported.

Fig. 4C shows the simulated digoxin transport inhibition curve by the same inhibitor with K_CQ_ = 1×10^6^ M^−1^, i.e. there is extensive hetero-dimerization. The NC plateau amplitude is the same, while less broad, but as the inhibitor concentration increases a sigmoidal decrease is seen down to essentially no digoxin transport. The free digoxin concentration at high inhibitor concentration is less than 10^−5^ µM, i.e. 4 orders of magnitude smaller than the initial concentration. So hetero-dimerization can also yield a sigmoidal curve, like inhibition of both the uptake transporter and P-gp, or inhibition of P-gp alone, but the complete inhibition PC plateau must be essentially no digoxin transport. In fact, the IC_50_ of the simulated inhibition curve is about 1 µM, i.e. the inverse of the K_CQ_ value for this simulation. This shows transport inhibition is essentially entirely due to the hetero-dimerization in this simulated extreme case.

This gives a reasonable criterion for distinguishing whether the non-P-gp inhibition is due to hetero-dimerization, hypothesis 3. If the inhibition curve is not sigmoidal, then the non-P-gp inhibition due to hetero-dimerization between the basolateral uptake transporter and inhibitor cannot be excluded. If the large inhibitor concentrations plateau, PC plateau, is significantly larger than zero, then hypothesis 3 is unlikely.

Since the inhibition curve for GF120918 in the MDCKII-MDRI (NKI) cells shows a PC plateau above zero, Fig. 3, hypothesis 3 is unlikely for these data. Thus this data supports the mechanism of inhibitor binding to the digoxin uptake transporter, i.e. hypothesis 1.

One more control was done, which was the inhibition of digoxin transport in these cells by nicardipine up to 100 µM. The inhibition curve was sigmoidal and the PC plateau was significantly above zero (data not shown). So nicardipine inhibition was due to binding to P-gp and not due to binding to digoxin. This compound was identified as forming colloidal aggregates at about 30 µM [18] [19] [20]. Clearly there is no binding of digoxin to nicardipine, so either these aggregates do not partition digoxin or they do not form in this media.

### Inhibition of digoxin transport by GF120918 in MDCK-MDR1 (NIH), Caco-2 and CPT-B2 cells

Our primary goal for these inhibition studies is to investigate whether a basolateral uptake transport mechanism also exists in other additional cell lines. The inhibition assays were performed with confluent cell monolayers of the respective cell lines, using digoxin as probe substrate at a concentration of 10 µM. The amount of digoxin transported in the B>A direction in 1 hr was measured in triplicate.

For fitting of the IC_50_ data in these additional cell lines, we assume that the P-gp specific kinetic parameters are system independent, i.e. equal to the values shown in Table 2. Preliminary work showed that the inhibition data could not be fitted solely by P-gp specific kinetic parameters for the probe substrate (k_1_, k_r_, and k_2_). We also have preliminary data that these P-gp specific rate constants extrapolate well between MDCK-MDR1-NKI cells and Caco-2 cells (Meng, Ellens & Bentz, unpublished), so it is likely that the elementary rate constants for P-gp expressed in the additional cell lines are well estimated by the values in Table 2. If digoxin transport across these cell lines involves only digoxin passive permeability and P-gp mediated efflux (i.e. is described entirely by passive permeability, P-gp elementary rate constants and P-gp surface density), then we should be able to fit the inhibition data with the elementary rate constants for digoxin and the inhibitor from Table 2, with Pgp surface density as a variable, as described below.

For simplicity and clarity, we define the efflux active P-gp surface density of a cell line relative to the number (800 P-gp/µm^2^) we have rigorously fitted for the MDCK-MDR1-NKI cell line, Table 2, and denote the fraction as T_rel_. So when we fit the efflux active P-gp surface density for the MDCK-MDR1-NIH cell line to be 60% of the value of T(0) shown in Table 2 for the MDCK-MDR1-NKI, then T_rel_ = 0.6.

We first fitted the P-gp surface density on the three additional cell lines relative to that on the MDCK-MDR1-NKI cell line by using the average digoxin transport at the lowest inhibitor concentration in each cell line (Table 3). We found that the surface density on the MDCK-MDR1-NIH cells was somewhat smaller (P-gp-rel of 0.6) and that the surface density on the Caco-2 cells and the CPT-B2 cells were substantially lower (T_rel_ = 0.2 and 0.3, respectively). Using Western blot analysis, it has been reported that Caco-2 cells express similar lower levels of P-gp than the MDCK-MDR1-NKI cells [10]. The efflux active P-gp level can be quite different between cell lines since, while it depends on P-gp expression levels, it also depends upon microvilli morphology, e.g. height of microvilli and distance between the microvilli in a complex way [5] [21] [22].

**Table 3:** Fitted parameter values of efflux active Pgp for each cell line relative to the MDCK-MDR1-NKI line and K_QB_ for the digoxin basolateral transporter from digoxin transport inhibition.

Cells	T_rel_ a	K_QB_ (M^−1^)^b^			
		GF120918	CsA	KCZ	VRP
MDCK-MDR1-NKI	1.0	(2±1)×10^8^	N/D	N/D	N/D
MDCK-MDR1-NIH	0.6	(1±0.5)×10^7^	(3±2)×10^6^	(4±2)×10^5^	(7±3)×10^4^
Caco-2	0.2	(2±1)×10^7^	(2±1)×10^6^	(2±1)×10^6^	(3±1)×10^5^
CPT-B2	0.3	(2±0.7)×10^7^	(2±0.5)×10^6^	(4±2)×10^6^	(1±0.5)×10^6^

a The surface density of efflux active P-gp relative to MDCK-MDR1-NKI cells, Table 2, is fixed by the average of the amplitudes of the inhibition curves for each cell line at the lowest inhibitor concentration. No error bars are given since these values were simply fitted to the digoxin concentration at the lowest inhibitor concentration by a single digit value.

b The “effective” binding parameter of the inhibitors to a basolateral transporter which block digoxin transport. Error bars were taken as single digit values to the fits.

**Figure 5:**
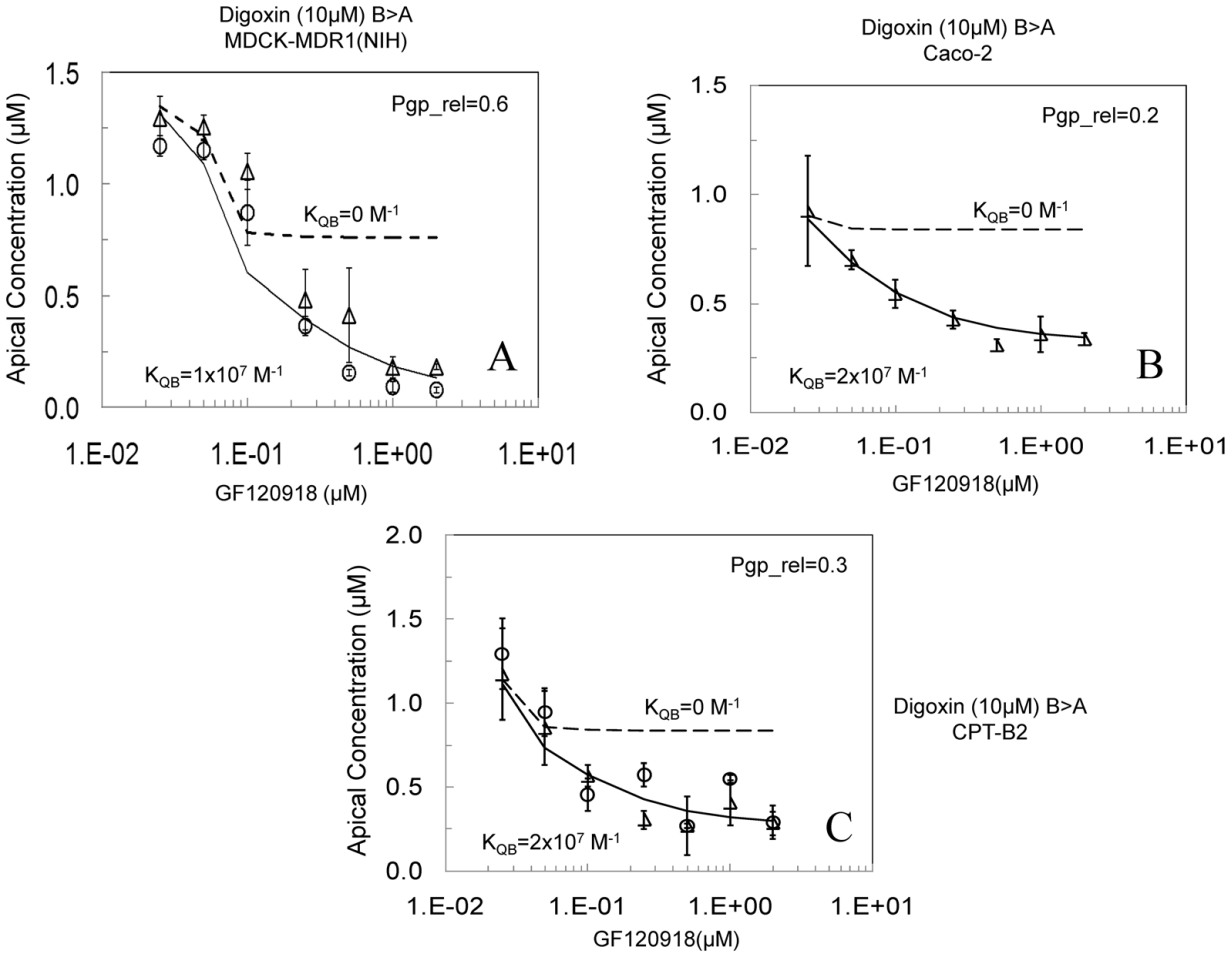
Inhibition of 10 µM digoxin B>A transport across cell monolayers, as measured on the apical side after 1 hr with increasing concentrations of GF120918 using the MDCK-MDR1-NIH cell monolayers (Fig. 5A), Caco-2 monolayers (Fig. 5B), and CPT-B2 monolayers (Fig. 5C), data acquired at Absorption Systems. Open symbols represent experimental data sets. The dotted lines shows the fitted inhibition assuming that the inhibitor binds only to P-gp, i.e. K_QB_ = 0. The solid lines show the fit using the values of K_QB_ shown in Table 3.

Symbols in Fig. 5A show two independent data sets for the inhibition of digoxin transport by GF120198 for the MDCK-MDR1-NIH cells, with error bars showing their standard deviation. The predicted inhibition of digoxin transport when T_rel_ = 0.6 and only P-gp is inhibited is shown by the dashed line. While the smaller inhibitor concentrations fit well enough, when [GF120918]>0.1 µM there was no fit. Much more inhibition was observed then was predicted by P-gp inhibition alone, indicating a GF120918 inhibitable digoxin uptake mechanism. A good fit to all the data was observed when T_rel_ = 0.6 and hypothesis 1 was tested with a K_QB_ = 1×10^7^ M^−1^ (solid line). While the fitted K_QB_ for GF120918 is larger in the MDCK-MDR1-NKI cell line, we do not know whether the basolateral digoxin uptake transporters in these two cell lines are related or not. Even if they are the same transporter, their surface densities could be quite different.

Similar results were obtained with the Caco-2 cell line and its BCRP knockdown line (CPT-B2). Fig. 5B shows the inhibition of 10 µM digoxin transport through the Caco-2 cell line at increasing concentrations of GF120918. To fit the amplitude of the data at the lowest GF120918 concentrations, T_rel_ = 0.2 for the Caco-2 cells. The dashed curve shows the simulation when GF120918 does not bind to the basolateral transporter, i.e. K_QB_ = 0 M^−1^ and the solid line shows the simulation for hypothesis 1 when GF120918 binds to the basolateral transporter with K_QB_ = 2×10^7^ M^−1^.

Fig. 5C shows the inhibition of 10 µM digoxin transport through the CPT-B2 cell line, at increasing concentrations of GF120918. To fit the amplitude of the data at lowest GF120918 concentrations, T_rel_ was fixed at 0.3. The dashed curve shows the simulation when GF120918 does not bind to the basolateral transporter, K_QB_ = 0 M^−1^ and the solid line shows the simulation for hypothesis 1 when GF120918 binds to the basolateral transporter with K_QB_ = 2×10^7^ M^−1^.

Hypothesis 3 is unlikely, since the PC plateaus are all above zero and because there is 10 µM of digoxin and less than 2 µM GF120918. Hypothesis 2 is unlikely for the same reasons as given with Fig. 3. Thus GF120918 non-P-gp inhibition is due to GF120918 binding to the basolateral uptake transporter. This means that each of these cell lines must be presumed to express a basolateral uptake transporter that is inhibited by GF120918.

### Inhibition of digoxin transport by CsA, ketoconazole (KCZ) and verapamil (VRP) in MDCK-MDR1 (NIH), Caco-2 and CPT-B2 cells

**Figure 6:**
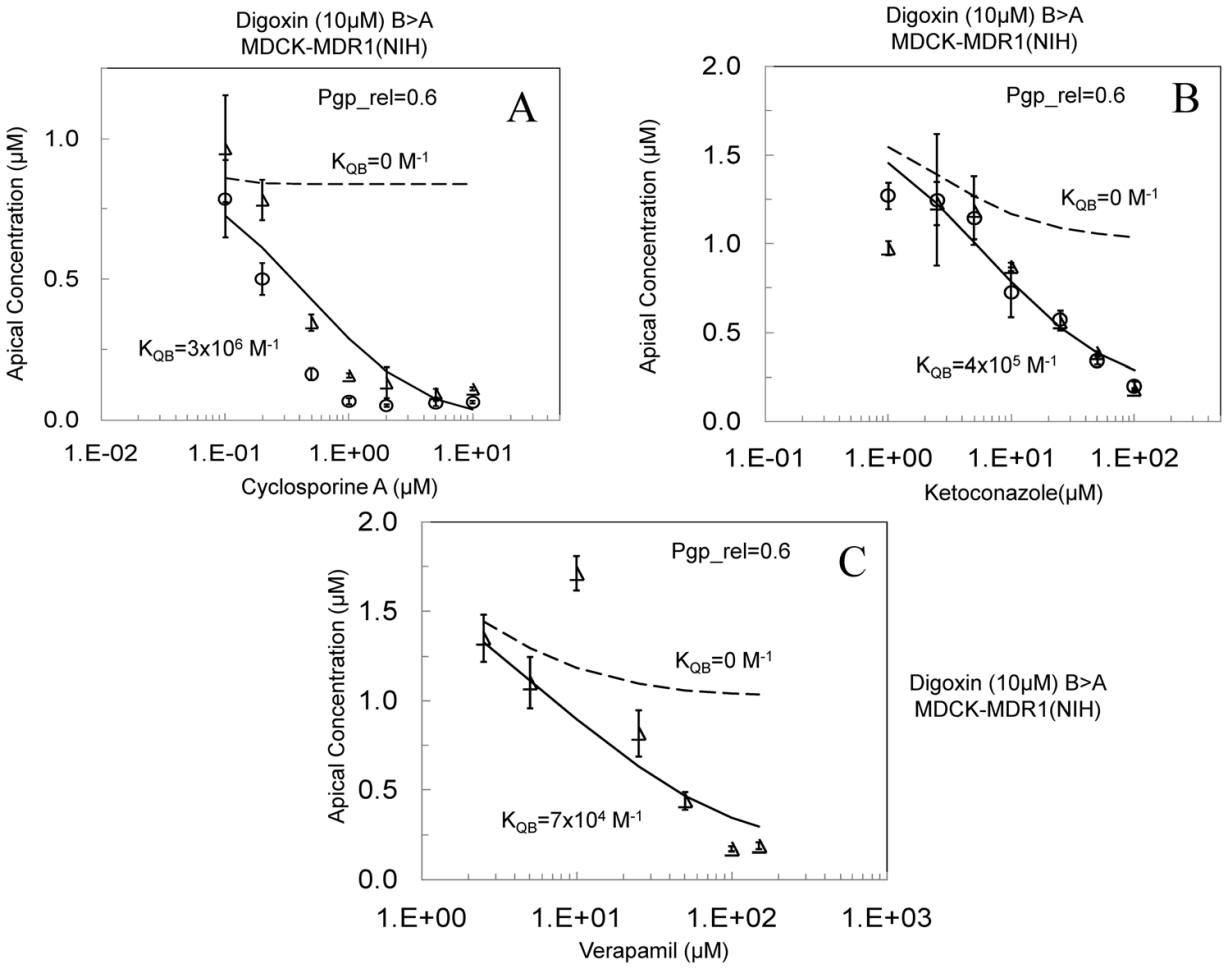
Inhibition of 10 µM digoxin B>A transport across the MDCK-MDR1-NIH cell monolayers, as measured on the apical side after 1 hr with increasing concentrations of cyclosporine A (Fig. 6A), ketoconazole (Fig. 6B) and verapamil (Fig. 6C), data acquired at Absorption Systems. Open symbols represent experimental data sets. The dotted lines shows the fitted inhibition assuming that the inhibitor binds only to P-gp, i.e. K_QB_ = 0. The solid lines show the fit using the values of K_QB_ shown in Table 3.

Fig. 6 examines the MDCK-MDR1-NIH cells with the other inhibitors. For all these fits, T_rel_ was fixed at 0.6 (Table 3), since this value must be the same in each experiment using that cell line. The dashed line shows the predicted inhibition for just P-gp, K_QB_ = 0, and the solid line shows the fit for hypothesis 1 using the K_QB_ values in Table 3. Fig. 6A with cyclosporine-A, CsA, shows substantial non-P-gp inhibition at 1 µM CsA and the PC plateau is well above zero. So, hypothesis 3 is unlikely. We note that with CsA's partition coefficient K_BO_ = 300 and 1 µM CsA, Eq. 4 would predict there would be about 0.5 CsA molecule per 1000 lipids, making hypothesis 2 even more unlikely for CsA than it was for GF120918. Thus, the non-P-gp inhibition by CsA appears to be due to its binding to the basolateral digoxin uptake transporter. This gives a second example showing the kinetic need for a basolateral uptake transporter in all these cells that is inhibited by GF120918 and CsA.

Fig. 6B with ketoconazole, KCZ, shows that the KCZ complete inhibition plateau was not reached, so both Hypothesis 1 and 3 remain as possibities. Other data for KCZ with other cells was also inconclusive. KCZ is not known to form the large aggregates with itself [31] [32] [33]. We note that with KCZ's partition coefficient K_PC_ = 400 and 100 µM KCZ showing substantial non-P-gp inhibition, there would be about 60–70 KCZ per 1000 lipids. This is not a miniscule number. However, from the data shown in Fig. 2B, the passive permeabilities, +GF120918, at 2 h are roughly the same from 1–30 µM KCZ, average over the 4 concentrations is (1.2±0.1)×10^3^ nm/s. Thus, there was no significant physical change in the plasma membrane permeability by KCZ, so hypothesis 2 is unlikely.

Fig. 6C with verapamil, VRP, shows that the VRP PC plateau at 100 and 150 µM was above zero, but the curve is ambiguous, so hypothesis 3 remains a possibility. Whether VRP forms aggregates or not has not been published, but it is very water-soluble. We note that with VRP's partition coefficient K_PC_ = 300 and 100 µM VRP, with perhaps complete non-P-gp inhibition, there would be about 50 VRP per 1000 lipids. This is not a miniscule number. However, from the data shown in Fig. 2C, the passive permeabilities, +GF120918, at 2 h are roughly the same from 1–30 µM VRP, average over the 4 concentrations is (7±2)×10^2^ nm/s. Notably, the permeability at 1 µM VRP was the smallest, in contrast to the prediction of hypothesis 2. It is hard to imagine how either of these drugs could physically alter the membrane so as to reduce digoxin's passive permeability substantially, while leaving their own passive permeabilities unchanged. Thus, there was no evidence of significant physical change in the plasma membrane permeability, so hypothesis 2 is unlikely.

Finally, the fitted K_QB_ values typically vary by inhibitor and by cell line. With respect to hypothesis 1, this would be expected, since K_QB_ would depend upon the expression level of BT, the binding constant of the inhibitor to BT and, if the binding site were within the membrane, the partition coefficient of the inhibitor to the basolateral membrane. With respect to hypothesis 2, K_QB_ would depend upon the partition coefficient of the inhibitor to the basolateral membrane, K_BO_, and an activity coefficient relating the mol fraction of inhibitor in the membrane, X_Q_ in Eq. 4, to the reduction in the passive permeability coefficient. Given the lack of support for hypothesis 2 by the data thus far, speculation about the form of an unknown activity coefficient is unwarrented. With respect to hypothesis 3, K_QB_ would be independent of the cells, which is only the case for CsA and is likely coincidental. The variation of K_QB_ values over the cell lines is greatest for KCZ and VRP, making hypothesis 3 unlikely for these two inhibitors. This offsets the ambiguity from the data in Fig. 6B anvf d 6C. So, the fitted K_QB_ values support only hypothesis 1.

### Passive permeability versus uptake transport

We noted that compounds with a passive permeability higher than amprenavir (quinidine, verapamil, ketoconazole) did not have a kinetic need for a basolateral uptake transporter. We therefore investigated another compound, vinblastine, with a low passive permeability, close to that of digoxin, for it's kinetic need for an uptake transporter. We note that while CsA has a low passive permeability, its efflux transport is so small, Fig. 2C, because k_2_ is so small, that a reasonable evaluation of its kinetic need for a transporter is not possible.

**Figure 7:**
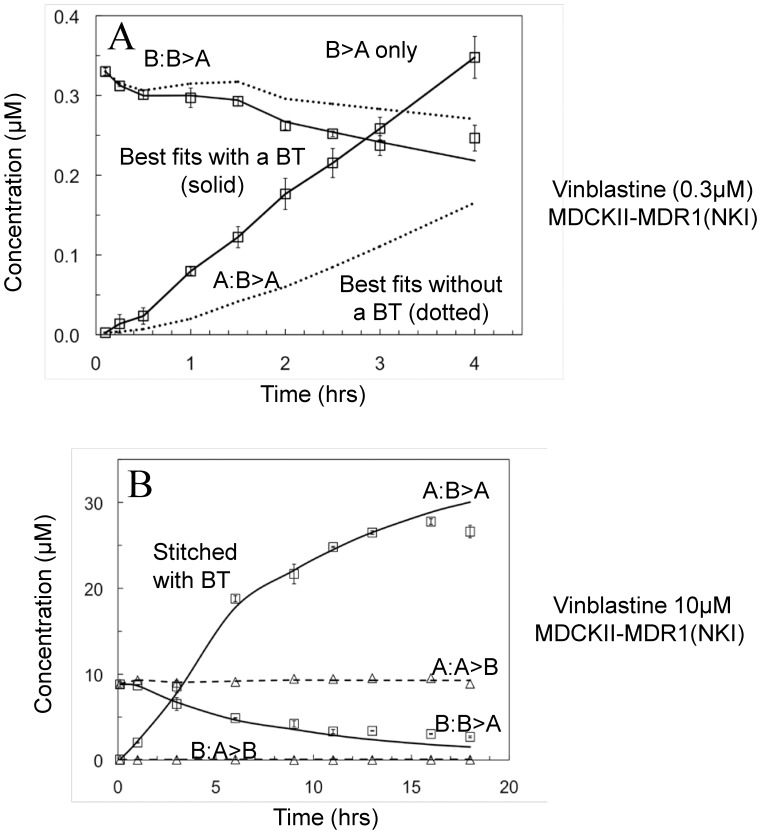
Transport data of vinblastine and their fitted curves across the MDCK-MDR1-NKI cell monolayers, data acquired at GSK. The same nomenclature is used as in Fig. 2. Fig. 7A shows the 6 hour experiment for 0.3 µM vinblastine. The dotted line shows the best fit with just P-gp inhibition. With BT, a good fit could be obtained, but not a unique fit, since the transport curves were essentially straight. Fig. 7B shows a stitched 18 hour experiment for 10 µM vinblastine, which was required to get unique fits for the kinetic parameters. The fitted parameters are shown in Table 2.

We performed a series of bidirectional transport studies with radiolabelled vinblastine (initial donor chamber concentrations of 0.003, 0.01, 0.03, 0.1, 0.3, 1, 3, 10, 30 µM). The vinblastine data could not be well fitted by P-gp alone. The dotted line above the B∶A>B data and below the A∶B>A data in Fig. 7A shows the “best” fit by P-gp alone, which used irreversible binding of vinblastine to P-gp and the maximum possible efflux rate constant of k_2_ = 100 s^−1^. The problem is that vinblastine's passive permeability in the presence of GF120918 was so low that the measured transport in the absence of GF120918 could not be achieved by the fits, like digoxin and loperamide [12]. Adding a basolateral uptake transporter gave the fit shown by solid lines through the data in Fig. 7A.

Because the vinblastine transport was essentially linear in time up to 4 hours, the fit shown in Fig. 7A can be obtained by an infinitely wide range of k_r_ and k_2_ pairs. Curvature in the transport data over time is required to obtain unique fits [13]. Curvature shows that the system is reaching the steady-state where the P-gp efflux out of the cells into the apical chamber equals the passive permeability into the cell from the apical chamber.

To obtain data with curvature for vinblastine transport, we must monitor the transport over a much longer period of time, as we have done previously for digoxin [12] [13]. We performed this experiment at a single larger concentration of 10 µM vinblastine to approach steady state within a reasonable time frame. To avoid problems with cell viability [12], the experiment was performed in 3 intervals of 6 hours each. Experiment 1 measured B>A and A>B transport of 10 µM vinblastine over the first 6 hours. The vinblastine concentrations measured at 6 hours in the donor and receiver compartments are then used as the starting concentrations for Experiment 2 to measure transport for the 6–12 hours interval. This was repeated for Experiment 3 for the 12–18 hours interval. These data were stitched together to generate transport data over 18 hours. The solid line in Fig. 7B is the fit to the data with incorporation of a basolateral uptake transporter. These fitted rate constants are reported in Table 2.

We have added vinblastine to the list of drugs transported by a basolateral uptake transporter in the MDCK-MDR1-NKI cells. Given that both digoxin and vinblastine have passive permeabilities <100 nm/s, i.e. it would take over a week for passive permeability to achieve 50% of the final steady-state concentration in the receiver chamber [5], it is easy to expect that an uptake transporter would be involved in their transport. On the other hand, loperamide requires a basolateral uptake transporter to obtain good fits to the data at low concentrations and has a passive permeability of ∼320 nm/s, which does not appear to be significantly lower than that of amprenavir at ∼350 nm/s, which shows no kinetic need a for a basolateral transporter. This raised a question of how low the passive permeability would have to be before we could observe the kinetic need for an uptake transporter from fitting the data.

**Figure 8:**
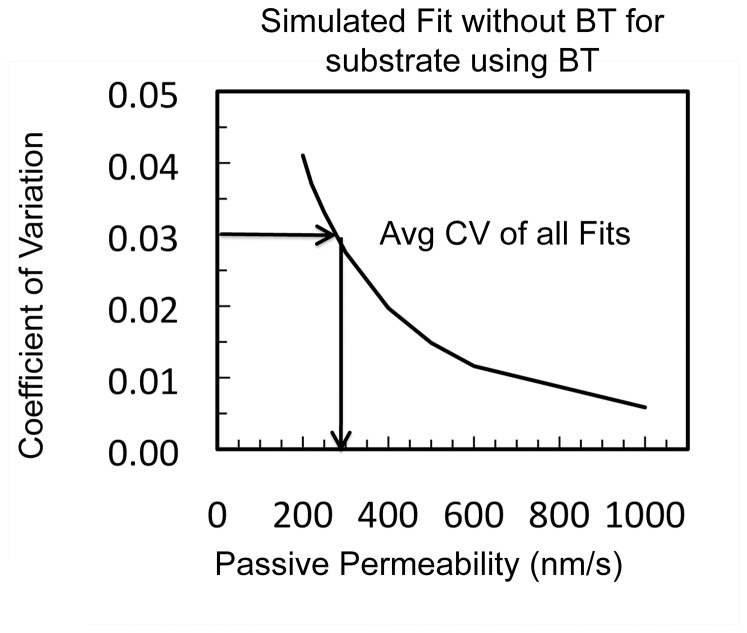
Simulation of +GF120918 passive permeabilities where the kinetic necessity of a basolateral digoxin uptake transporter could be validated. Simulated transport data was created across the MDCK-MDR1-NKI cells for a virtual hybrid molecule with the P-gp rate constants of verapamil and the basolateral transporter clearance of loperamide (k_A_ = 0, k_B_ = 100 s^−1^). The passive permeability was varied between 200–1000 nm/s. The substrate concentration range used for fitting was 3 nM to 30 µM. The simulated data were analyzed using only P-gp, i.e. only k_r_ and k_2_ were used to fit the data. The coefficient of variation, CV, for the best fit at each passive permeability value was obtained. When the fits deteriorated to a coefficient of variation, CV, above 0.03, or 3%, the kinetic need for the basolateral digoxin uptake transporter becomes plausible [13].

We addressed this question using simulations of a virtual drug. We simulated a P-gp substrate using the kinetic parameters of verapamil in the MDCK-MDR1-NKI cell line in Table 2, with the added properties that we varied it's passive permeability and it used a basolateral uptake transporter with a k_B_ = 100 s^−1^, i.e. loperamide's value. We then fitted these simulations assuming that P-gp was the only transporter involved. The result is plotted on Fig. 8. Clearly, as the passive permeability got smaller, the fit constrained to using only P-gp got worse, especially at the smaller substrate concentrations. Our current benchmark for fits that may require another transporter is a coefficient of variation CV>0.03 [13]. Interestingly, it turned out that a passive permeability between those of loperamide and amprenavir marked this transition, Fig. 8.

## Discussion

Digoxin is a widely prescribed cardiovascular drug with a narrow therapeutic index and therefore not very tolerant of drug-drug interactions. Since digoxin is a P-gp substrate, digoxin drug-drug interactions are often ascribed to P-gp inhibition. It is common practice in the pharmaceutical industry to assess the risk for a clinically relevant digoxin drug-drug interaction by determining the in vitro P-gp inhibitory potency using P-gp expressing cell lines [1] [8] [11]. We have demonstrated kinetically that digoxin transport across MDCK-MDR1-NKI cells involves a basolateral and an apical uptake transporter as well as P-gp, although we were not able to determine the identity of that transporter, other than that it was inhibited by GF120918 [12] [13]. Recently, it has been reported that digoxin is a substrate for uptake transporters in other cell lines as well [16] [17]. The involvement of other transporters besides P-gp in digoxin transport across P-gp expressing confluent monolayers in MDCK-MDR1-NKI cells raises two questions: (1) does the experimentally measured IC_50_ in these cells represent inhibition of P-gp, inhibition of the uptake transporters, or a combination of the two and (2) do other P-gp expressing cell lines also express a digoxin uptake transporter.

To investigate these questions, we used three other cell lines: MDCK-MDR1-NIH, Caco-2 and CPT-B2, which is the Caco-2 cell with a BCRP knockdown. Digoxin B>A transport across these cells was inhibited by four inhibitors: GF120918, cyclosporine-A (CsA), ketoconazole (KCZ) and verapamil (VRP). The inhibition curves were analyzed to answer two questions: (1) Can the curve be explained solely by the inhibition of P-gp and if not, (2) what is the source of the non-P-gp inhibition portion of the curve. Using our kinetic analysis based upon fitting elementary rate constants, rather than Michaelis-Menten steady-state parameters, we found that in all cases there was substantial non-P-gp inhibition.

The next question was that of mechanism. We have examined three different hypotheses to identify the source of the non-P-gp inhibition:

1. The cell line has a basolateral digoxin uptake transporter that is inhibited.
2. Partitioning of inhibitor into the basolateral membrane causes the membrane to become less permeable.
3. There is hetero-dimerization of digoxin with the inhibitor in the basolateral donor chamber that reduces the free digoxin concentration, thereby inhibiting digoxin uptake into the cell.

Hypothesis 1 assumes that the cell line has a basolateral digoxin uptake transporter, which has only been clearly implicated for the MDCK-MDR1-NKI cell line [12], and the P-gp inhibitor can bind to it. We have found that this model can fit all of the data. Furthermore, for GF120918 and CsA, hypotheses 2 and 3 were unlikely for all cell lines, suggesting that all cell lines do have a kinetically identified basolateral digoxin uptake transporter.

Hypothesis 2 appears simplest because nothing beyond partitioning of the inhibitor into the basolateral membrane is required, which is certain, but the mechanism of permeability reduction is unspecified. We found there was substantial non-P-gp inhibition at concentrations of CsA where the measured partition coefficient, K_BO_.

In Table 1, would predict about 0.5 CsA molecule per 1000 lipid molecules, Fig. 6A. We could not measure a partition coefficient for GF120918 since no radiolabelled compound is available. However, if we assume a partition coefficient of 10^4^, which is 20-fold larger than any partition coefficient measured in Table 1, then we found substantial non-P-gp inhibition at GF120918 concentrations where we would predict about 2 GF120918 molecules per 1000 lipid molecules, Fig. 3. Such miniscule concentrations in the membrane would not diminish digoxin permeability by any known mechanism.

For KCZ and VRP, we found substantial non-P-gp inhibition at concentrations where the measured partition coefficients, Table 1, would predict about 40–70 inhibitor molecules per 1000 lipid molecules, Fig. 6B & 6C. This is not a miniscule concentration in the membrane. However, the passive permeabilities of both drugs were basically stable at concentrations ranging from 1–30 µM, Figs. 2A & 2B. It is hard to imagine how either of these drugs could reduce digoxin's passive permeability so substantially, while leaving their own passive permeabilities unchanged. Thus, there was no evidence of significant physical change in the plasma membrane permeability in the presence of these drugs, so hypothesis 2 was unlikely.

Hypothesis 3 is a simplified model extrapolated from work showing that at least one P-gp substrate, nicardipine, can form micron sized aggregates [18] [19] [20], which theoretically could partition digoxin, thereby reducing its free concentration in the donor chamber. We have used a simpler model of hetero-dimerization between digoxin and the inhibitor, rather than dealing with an aggregate of inhibitor and a partition coefficient for digoxin into that aggregate. Both models yield the same qualitative behavior, i.e. the inhibition curves are the same with different parameter values (simulations not shown) and the hetero-dimerization is conceptually simpler.

Hypothesis 3 would have the inhibitor reduce digoxin uptake by hetero-dimerization, so that the free digoxin concentration would go to zero. By simulations, Fig. 4, we found criterion by which hypothesis 3 proved unlikely for each inhibitor. If the high inhibitor concentration plateau, i.e. PC plateau, is significantly above zero, then hypothesis 2 would be unlikely for that inhibitor.

Since the inhibition curve for GF120918 in all the cells is sigmoidal and the PC plateau is above zero, Figs. 3 & 4, hypothesis 3 is unlikely for GF120918. Thus, for GF120918, only hypothesis 1 is supported by the data, i.e. there is a basolateral digoxin uptake transporter that is inhibited by GF120918 [12]. The same is true for CsA, Fig. 6A. The mechanism of non-P-gp inhibition is GF120918 and CsA binding to the digoxin uptake transporter in all cell lines, as proposed in Acharya et al. [12] for the MDCK-MDR1-NKI cells. Most importantly, this establishes the kinetic need for a basolateral digoxin uptake transporter in all cell lines, regardless of whether all the inhibiters used here actually bind to it.

For KCZ and VRP, hypothesis 2 is unlikely, as discussed above. For these drugs, the PC plateau was not clearly articulated, Figs. 6B & 6C, so hypothesis 3 could contribute to this inhibition. Binding of KCZ and VRP to a basolateral digoxin uptake transporter could also fit the data, Figs. 6B & 6C. Further experiments are required to determine whether or not hetero-dimerization also plays a role in the non-P-gp inhibition by these drugs.

We have added vinblastine to the list of drugs transported by a basolateral uptake transporter in the MDCK-MDR1-NKI cells. Given that both digoxin and vinblastine have passive permeabilities <100 nm/s, i.e. it would take over a week for passive permeability to achieve 50% of the final steady-state concentration in the receiver chamber [5], it is easy to expect that an uptake transporter would be involved in their transport. On the other hand, loperamide requires a basolateral uptake transporter to obtain good fits to the data at low concentrations and has a passive permeability of ∼320 nm/s, which does not appear to be significantly lower than that of amprenavir at ∼350 nm/s, which shows no kinetic need a for a basolateral transporter. This raised a question of how low the passive permeability would have to be before we could observe the kinetic need for an uptake transporter from fitting the data.

We addressed this question using simulations of a virtual drug. We simulated a P-gp substrate using the kinetic parameters of verapamil in the MDCK-MDR1-NKI cell line, Tables 1 and 2, with the added properties that it used a basolateral uptake transporter with a k_B_ = 100 s^−1^, i.e. loperamide's value, and we varied it's passive permeability. We fitted these simulations assuming that P-gp was the only transporter involved. Our current working benchmark for fits that may require another transporter is a coefficient of variation CV>0.03 [13]. Interestingly, it turned out that a passive permeability between those of loperamide and amprenavir marked this transition, Fig. 8. At higher passive permeabilities, the fits appear too good to justify incorporation of another transporter. CsA is exempted from this criterion because its efflux rate is too small to show significant kinetic need, even if a basolateral uptake transporter transports it.

## Conclusion

**Figure 9:**
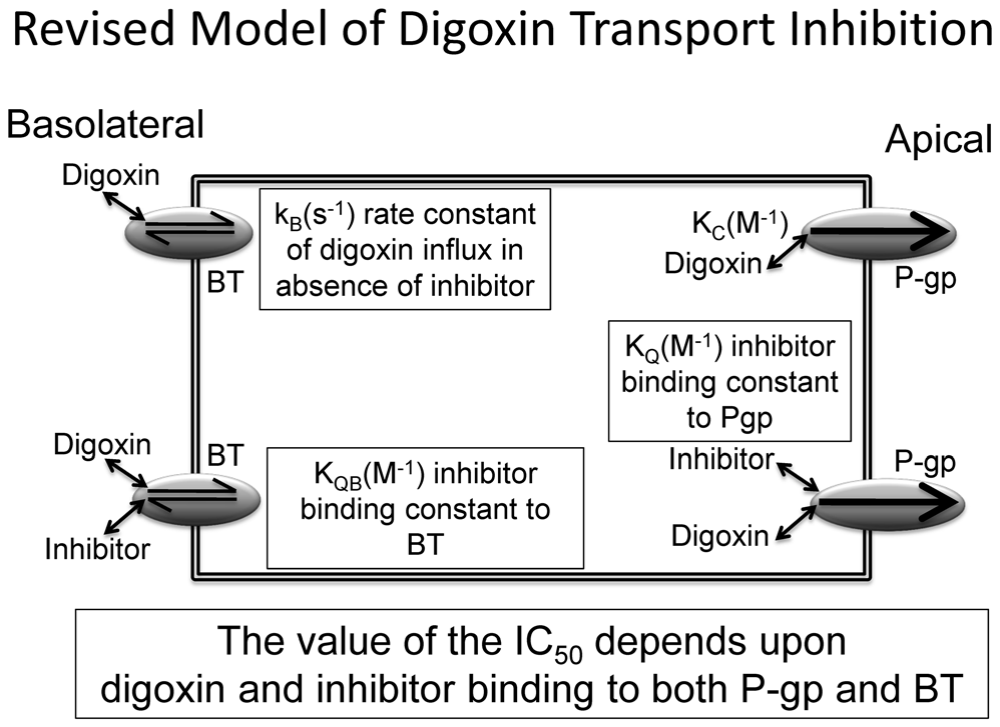
Model of transporters that are kinetically significant for digoxin transport and the inhibition of digoxin transport for all cell lines studied here. Starting from the basolateral chamber, the basolateral digoxin uptake transporter, denoted BT, transports digoxin into the cytosol more rapidly than bilayer permeability. This clearance is measured in the absence of inhibitor and denoted k_B_(s^−1^). Inhibitor binding to BT according to a binding constant denoted K_QB_ (M^−1^), defined in Eq. 4 and shown in Table 3, can inhibit this digoxin transport. Efflux of digoxin from the cell into the apical compartment is mediated by P-gp, according to the digoxin kinetic parameters, Table 2. This efflux can be inhibited by competitive binding of the inhibitor to P-gp, according to its binding constant K_Q_, which is equal to K_C_ in Table 2, since it is the inhibitor in this case.

The structural mass action kinetic analysis has been used to demonstrate that the IC_50_ for inhibition of digoxin transport across MDCK-MDR1-NKI, MDCK-MDR1-NIH, Caco-2 and CPT-B2 cells is a convolution of inhibition of P-gp and of a kinetically identified basolateral digoxin uptake mechanism. Three possible sources are discussed for this kinetically identified uptake mechanism: (1) inhibition of an uptake transporter, (2) inhibitor binding to the basolateral outer membrane directly reducing digoxin passive permeability and (3) aggregation between digoxin and inhibitor in basolateral chamber resulting in reduced free digoxin concentrations. The weight of evidence presented here supports the existence of a basolateral digoxin uptake transporter in all cell lines and that GF120918 and CsA can bind to this uptake transporter, inhibiting digoxin uptake by the cells. This model is shown in Fig. 9. A contribution from hetero-dimerization model cannot be completely excluded for ketoconazole and verapamil by these data. Further studies are required. Without this mass action kinetic analysis we developed using the MDCK-MDR1-NKI cells yielding elementary rate constants, the deconvolution of the inhibition curves shown here, the kinetic evidence of the existence of widely expressed basolateral transporters and the inhibition of these basolateral transports by some, if not all, P-gp substrates could not have been accomplished. We believe this kinetic analysis, with its powerful diagnostic tests for the kinetic requirement of other transporters, will continue to contribute to the current discussion on the relative importance of transporters and passive permeability in transport biology [31] [32] [33] [34]. Our work supports the importance of transporters and of passive permeability by providing a direct analytical kinetic method to measure their relative contributions simultaneously.

## Supporting Information

File S1 **Appendix S1**, Mass Action differential equations for the kinetic model for transport from the mass action reactions shown in Eqs. (1) and (2) are shown. **Glossary S1,** Definitions of variables and parameters used in the Mass Action equations shown in Appendix S1.
(DOCX)
