# Supplementary material for: Transport Inhibition of Digoxin Using Several Common P-gp Expressing Cell Lines Is Not Necessarily Reporting Only on Inhibitor Binding to P-gp
Source: PLoS One. 2013 Aug 16;8(8):e69394. doi: 10.1371/journal.pone.0069394 (PMC3745465; doi:10.1371/journal.pone.0069394)
Supplement: File S1 — Appendix S1, Mass Action differential equations for the kinetic model for transport from the mass action reactions shown in Eqs. (1) and (2) are shown. Glossary S1, Definitions of variables and parameters used in the Mass Action equations shown in Appendix S1. (DOCX) [file pone.0069394.s001.docx]

# Supplementary Information for

# Transport inhibition of digoxin using several common P-gp expressing cell lines is not necessarily reporting only on inhibitor binding to P-gp.

Annie Albin Lumen^#^, Libin Li^#^, Jiben Li, Zeba Ahmed, Zhou Meng, Albert Owen, Harma Ellens*, Ismael J. Hidalgo* & Joe Bentz*.

File S1. Appendix

Differential equations of the kinetic model for transport.

The mass action reactions shown in Eqs. (1) and (2) yield the following mass action equations. The parameters and variables are defined below in a Glossary.

 (S.1)

File S1. Glossary

Definitions of variables and parameters used in the Mass Action equations shown in Appendix S1. Unless otherwise specified, units for all concentrations are molar, either in the aqueous media or within the lipid bilayer, all times are seconds and all partition coefficients are [mols drug/L(lipid)]/ [mols drug/L(aqueous buffer)].

A Area of the Transwell insert (=1.13cm^2^)

A_A_ Area of the apical membrane (=2A)

A_B_ Area of the basolateral membrane (=2A)

C_A_ concentration of substrate in apical chamber

C_B_ concentration of substrate in basolateral chamber

C_C_ concentration of substrate in cytosol

C_PC_ concentration of substrate in cytosolic face of plasma membrane (=K_PC_C_C_)

K_AO_ equilibrium partition coefficient for substrate from the apical chamber to the apical membrane outer monolayer

K_BO_ equilibrium partition coefficient for substrate from the basolateral chamber to the basolateral membrane outer monolayer

K_PC_ equilibrium partition coefficient for substrate from the cytosol to the plasma cytosolic monolayer

K_QAO_ equilibrium partition coefficient for inhibitor from the apical chamber to the apical membrane outer monolayer

K_QBO_ equilibrium partition coefficient for inhibitor from the basolateral chamber to the basolateral membrane outer monolayer

K_QPC_ equilibrium partition coefficient for inhibitor from the cytosol to the plasma cytosolic monolayer

k_1_ second order rate constant for substrate binding to P-gp from inner plasma membrane (M^-1^s^-1^)

k_1Q_ second order rate constant for inhibitor binding to P-gp from inner plasma membrane (M^-1^s^-1^), assumed equal to k_1_.

k_2_ first order rate constant for substrate efflux from P-gp to the apical chamber (s^-1^)

k_2Q_ first order rate constant for inhibitor efflux from P-gp to the apical chamber (s^-1^)

k_A_ first order rate constant for the apical uptake transporter.

k_B_ first order rate constant for the basolateral uptake transporter.

k_r_ first order rate constant for substrate dissociation from P-gp back into the inner plasma membrane (s^-1^)

k_rQ_ first order rate constant for inhibitor dissociation from P-gp back into the inner plasma membrane (s^-1^)

k_v_ first order rate constant for substrate loss from aqueous chambers, e.g. mass balance (s^-1^)

P_BA_ +GF120918 permeability coefficient across the confluent monolayer from basolateral to apical chambers, equals B>A permeability divided by 2A (nm/s)

P_AB_ +GF120918 measured permeability coefficient across the confluent monolayer from apical to basolateral chambers, equals A>B permeability divided by 2A (nm/s)

P_BC_ +GF120918 permeability coefficient across the basolateral membrane to the cytosol (nm/s). P_BC_ is defined equal to P_BA_

P_AC_ +GF120918 permeability coefficient across the apical membrane to the cytosol (nm/s). P_AC_ is defined equal to P_AB_

[P-gp] total concentration of transporter in apical membrane inner monolayer

Q_A,B or C_ inhibitor concentration in the basolateral, cytosolic or basolateral compartments of the confluent cell monolayer as a function of time or the true steady-state value derived from the mass action kinetics. We use Q instead of I, which is difficult to read in text and MATLAB code.

<Q> preincubation average inhibitor concentration in all compartments, usually assumed to be constant. This was not assumed in our simulations or experiments, where the chamber contents were refreshed with new inhibitor solutions, as well as the donor side probe-substrate.

T_0_ concentration of transporter with nothing bound.

T_C_ concentration of transporter with one bound substrate molecule

T_Q_ concentration of transporter with one bound inhibitor molecule

V_A_ volume of Costar Transwell apical chamber (0.5 mL)

V_B_ volume of Costar Transwell basolateral chamber (1.5 mL)

V_C_ volume of entire cell monolayer cytosol (roughly 1 μL)

 entire apical volume accessible to substrate, =
 entire basolateral volume accessible to substrate, =
 entire cytosolic volume accessible to substrate,=

V_AO_ volume of outer apical membrane facing the Costar apical chamber (0.5 nL used here)

V_BO_ volume of outer basolateral membrane facing the Costar basolateral chamber (0.5 nL used here)

V_PC_ volume of entire cell monolayer inner plasma membrane facing the cytosol (1 nL used here)
